# Non-steroidal anti-inflammatory drug induced intestinal stricturing: diaphragm disease

**DOI:** 10.1093/jscr/rjad489

**Published:** 2024-01-18

**Authors:** Nathan Johnson, Maseera Solkar, Rishabh Sehgal, Kallingal Riyad

**Affiliations:** John Goligher Colorectal Surgery Unit, St James's University Hospital, Leeds LS9 7TF, United Kingdom; John Goligher Colorectal Surgery Unit, St James's University Hospital, Leeds LS9 7TF, United Kingdom; John Goligher Colorectal Surgery Unit, St James's University Hospital, Leeds LS9 7TF, United Kingdom; John Goligher Colorectal Surgery Unit, St James's University Hospital, Leeds LS9 7TF, United Kingdom

**Keywords:** diaphragm disease, inflammatory bowel disease, NSAIDs, stricture

## Abstract

Diaphragm disease (DD) is a rare small bowel enteropathy associated with non-steroidal anti-inflammatory drug use. Since the first description there have only been approximately 100 cases of DD reported in the literature. Stricturing webs or ‘diaphragms’ form in the bowel, causing non-specific abdominal symptoms that can ultimately lead to bleeding and obstruction. Diagnosis is notoriously challenging as there is no single gold standard investigation. We present two cases of DD both of which were ultimately diagnosed by surgical resection. We also propose a novel flow algorithm that can be utilized for working up patients with suspected DD.

## Introduction

Non-steroidal anti-inflammatory drugs (NSAIDs) are one of the most commonly prescribed analgesics. In 2010, almost 17 million prescriptions were issued for NSAIDs in the UK. They are a highly effective drug class for the management of pain and inflammation. Unfortunately, the use of NSAIDs is associated with numerous adverse effects including gastrointestinal bleeding, cardiovascular side effects and nephrotoxicity. The development of NSAID associated enteropathy and diaphragms or webs causing vague nonspecific abdominal symptoms are rare. Lang *et al.* [1]*.* first used the term ‘diaphragm disease’ (DD) in 1988 to describe the pathologic findings of nonspecific small-bowel disease associated with NSAID use. Since then, there have only been approximately 100 cases of DD reported in the literature. This condition is notoriously difficult to diagnose and usually requires a multimodal approach and a high index of suspicion. Although the pathophysiology and natural history of DD has yet to be fully elucidated, surgery remains the mainstay of management. Herein we report two cases of DD presenting with a long history of non-specific abdominal symptoms.

## Case 1

A 41-year-old male was originally referred to the gastroenterology service with iron deficiency anaemia necessitating several blood and iron transfusions. His symptoms were investigated with a gastroscopy and colonoscopy, both of which failed to diagnose a cause. As such he underwent capsule endoscopy that led to the patency capsule becoming lodged in the mid-ileum. A follow-up computerized tomography (CT) scan of the abdomen and pelvis demonstrated non-specific 5 cm thickening in the mid-ileum, with Crohn’s disease being the top differential. His case was discussed at the gastrointestinal (GI) multidisciplinary team (MDT) meeting and the diagnosis was thought to be most likely in keeping with Crohn’s disease however this could not be certain. The options put forth to the patient were colonic approach enteroscopy, surgery or commence medical treatment for Crohn’s disease. The patient opted for colonic enteroscopy however this also failed to clarify the diagnosis. This case was re-discussed at the GI MDT and the patient was admitted electively for laparoscopy. This demonstrated multifocal short active inflammatory strictures, presumed at the time to be Crohn’s disease. The patient was discharged on tapering does of oral steroids. He was followed up in the inflammatory bowel disease (IBD) clinic and commenced on combination therapy with infliximab and azathioprine. Unfortunately, he did not respond to medical therapy as he remained anaemic with continued nonspecific intermittent abdominal pain. As he was thought to be a primary non-responder to infliximab he was then switched to adalimumab. A follow-up small bowel MRI demonstrated multifocal short fibrotic strictures, possible superficial terminal ileitis and left sided colitis. The patient was then reviewed in the combined IBD clinic and continued to be symptomatic while being on adalimumab. As he had exhausted all medical options, the consensus of the combined IBD clinic was to book the patient for surgery. The patient underwent a laparoscopic assisted small bowel resection and on table enteroscopy with formation of ileostomy and mucous fistula. Histopathology demonstrated small bowel with multiple web-like strictures, suggestive of DD (‘samples from the areas of macroscopically identified strictures in the small bowel showed mucosal ulcers with underlying fibrosis comprising vertically orientated fibres drawing up the muscle proper and creating web-like areas of stricturing’). There were no histological features of Crohn’s disease. The patient’s ileostomy was subsequently closed six months post-operatively. It transpired that the patient was intermittently self-medicating with over-the-counter preparations of ibuprofen and codeine for arthritic back pain for several years. His symptoms resolved on follow up once he stopped NSAID use.

## Case 2

**Figure 1:**
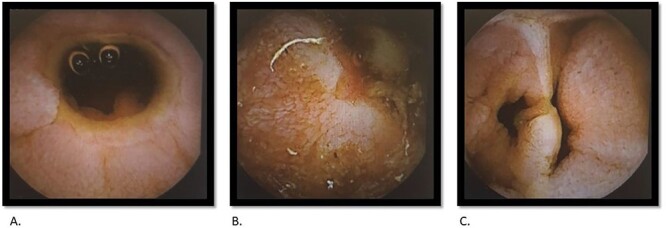
Capsule endoscopy: tight stricture noted within the ileum (A) with multiple areas of ulceration (B) and an area with apparent false lumen (C).

**Figure 2:**
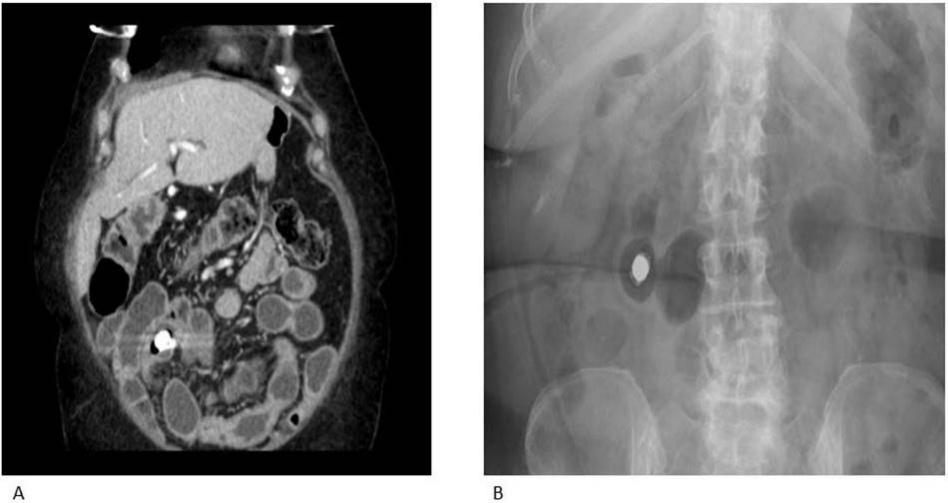
CT enterography (A) and plain X-ray (B) demonstrating multiple areas of mid small bowel stricturing. Some evidence of deep ulceration and skip lesions. Of note, the capsule remains stuck within the small bowel and is at risk of causing obstruction.

A 69-year-old female had previously undergone a sleeve gastrectomy and Roux-en-Y bypass. She was initially seen in the Haematology clinic with iron deficiency anaemia presumed secondary to her gastric bypass. Upper and lower GI endoscopy were within normal limits. She was managed with iron infusions. Of note, the patient had been on diclofenac for several years due to chronic back pain. Few months later she was reviewed in the cardiology clinic for shortness of breath on exertion and referred to gastroenterology due to her low albumin and haemoglobin. Repeat gastroscopy was normal and colonoscopy demonstrated ulcerations on the ileocaecal valve thought to be secondary to diclofenac use. She underwent a capsule enteroscopy that demonstrated a grossly abnormal distal ileum with a series of tight ulcerated strictures (Fig. 1). A CT of the abdomen and pelvis demonstrated multiple areas of mid small bowel stricturing and a further segment of terminal ileal abnormality. There was some evidence of deep ulceration and a diagnosis of Crohn’s disease remained on top of the list of differentials due to the presence of ulceration and skip lesions. The capsule from her enteroscopy was noted to be stuck within the small bowel (Fig. 2). The patient subsequently underwent ileo-caecectomy, on table stricture dilation and stricturoplasty. Histology demonstrated multiple small bowel ulcers with focal structuring and multiple small intramural diverticulae. No evidence of Crohn’s disease. NSAIDs were the most probable cause of the histologic appearance. The patient was advised to stop consuming NSAIDs and made a slow recovery thereafter.

## Discussion

In 1988, Lang *et al.* [1]. were the first to study operative small bowel resection specimens over a 16-year period to assess whether any intestinal disease could be directly attributable to NSAID use. A spectrum of patterns was found from multiple (4–70) pathognomonic ileal mucosal concentric rings of fibrotic tissue known as ‘diaphragms’ to broad strictures. Such thin walled (2–4 mm) diaphragms project into the bowel mucosa and can cause luminal narrowing leading to obstructive symptoms and perforation. The classical histological characteristic of a diaphragm is that of localized submucosal fibrosis and this distinguishes a diaphragm from exaggerated normal plicae within the bowel. Even though the overall prevalence of this rare condition is difficult to ascertain, Maiden *et al.* [2]. found a 2% incidence of diaphragm-like strictures within their cross-sectional capsule enteroscopy study cohort. DD is not exclusive to the small bowel as several authors have reported colonic DD. Munipalle *et al.* [3] recently performed a systematic review on colonic DD and included 45 cases. The highest incidence was in the seventh decade of life, with a female preponderance. Most patients presented with chronic (median 3 months) and multiple nonspecific gastrointestinal symptoms. The median time of NSAIDs consumed was 5 years with diclofenac being the most commonly used drug. Colonoscopy was the most informative investigation and the ascending colon was the most common site of DD. Nearly two-thirds of the patients were treated by discontinuing NSAID treatment combined with other forms of treatment including therapeutic endoscopic techniques and surgical resection.

The pathogenesis of small bowel injuries caused by NSAIDs has not been fully elucidated. Bjarnason *et al*. [4]. have suggested a three-hit hypothesis. Firstly the phospholipid in the cell membrane on the mucosal surface is directly damaged by NSAIDs via COX-1 inhibition leading to injury of the mitochondria. Secondly, mitochondrial damage leads to decreased energy synthesis resulting in calcium efflux and generation of free radicals. This leads to disruption of intercellular junctions and increased mucosal permeability. Thirdly, the intraluminal contents invade the cell through the weakened mucosal barrier leading to inflammation that propagates to diaphragm and stricture formation. Stricturing of the small bowel is commonly seen in Crohn’s disease with other less common differentials including ingestion of potassium chloride tablets, radiation exposure, anastomotic sites, tuberculosis, small and large bowel lymphomas, and low flow states. Thus, the diagnosis of DD requires the exclusion of other common causes of stricture formation.

The signs and symptoms of NSAID-induced enteropathy are usually nonspecific. They have various manifestations, such as iron-deficiency anaemia secondary to occult or overt gastrointestinal bleeding, protein loss leading to hypoalbuminemia, indigestion, constipation, diarrhoea and abdominal pain [5, 6]. Serious clinical complications such as life-threatening haemorrhage, obstruction and perforation occur infrequently.

Making a diagnosis of DD is challenging as demonstrated in the two cases outlined. Investigations usually provide non-specific or misleading results and definitive diagnosis is often only confirmed after surgical resection and histology. Blood tests (e.g. FBC, Ferritin, LFTs) are non-specific and usually only demonstrate an anaemia or poor nutritional state [7], merely indicating a need for further investigations. Plain film abdominal radiographs may show dilated loops of bowel in acutely obstructed patients and have very limited use otherwise. CT scans of the abdomen can highlight transition points in obstructed bowel or areas of stricturing disease however they cannot elucidate the underlying cause and the differentials for small bowel strictures are numerous. Oral contrast can also be administered with these scans however any diaphragm strictures present can easily be mistaken for valvulae conniventes [8, 9]. There are several reports in the literature of DD being diagnosed with capsule endoscopy [10, 11]. It is worth noting that in these cases the capsule was retained necessitated laparotomy and resection of the affected bowel. Therefore, to reduce the risk of causing acute obstruction in higher risk patients it may be worth testing the patency of the bowel with a dissolvable capsule initially. Double balloon enteroscopy (DBE) allows for visualization of the entire small and large bowel, and hence can be used to identify DD strictures and obtain biopsies [12]. However DBE requires a specialist endoscopist and evidence demonstrates that it has similar diagnostic yields to capsule endoscopy when investigating small bowel disease [13]. Whilst bleeding from DD lesions can occur, the rate is often too slow to be detected on dedicated imaging such as isotope-labelled red cell scanning and angiography [9, 14]. Diagnostic laparoscopy can be attempted in cases of suspected DD, however the serosal surface of the bowel often appears normal and this makes it difficult to identify the sites of diaphragm strictures [11, 15]. Laparotomy and palpation of the entire length of small bowel is superior as it allows for identification of all sites of diaphragm stricturing [11, 15, 16]. Subsequent resection of the affected segments and histological examination to confirm the diagnosis can then be carried out.

**Figure 3:**
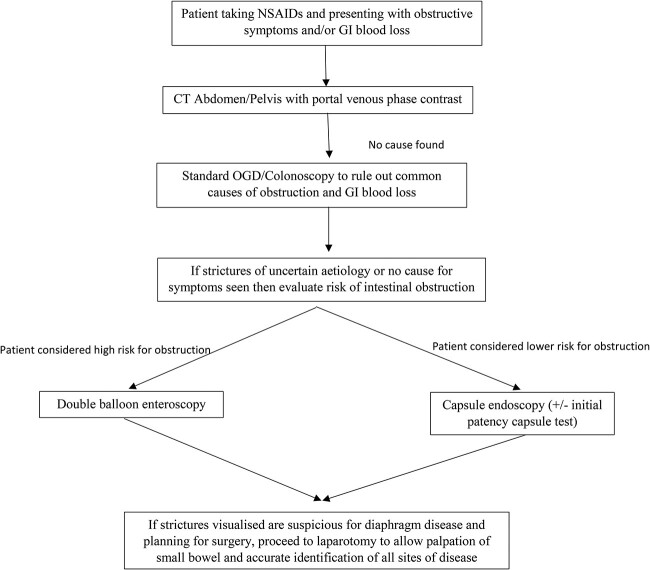
Flow diaphragm outlining the investigations of a potential patient with DD.

Based on our own experience and a review of the available literature, we have proposed a flow diaphragm (Fig. 3) outlining the investigation of a potential patient with DD.

## Conclusion

DD is a condition that is difficult to diagnose and a high index of suspicion is required in patients taking NSAIDs and presenting with anaemia with associated intermittent gastrointestinal obstructive type symptomatology. Surgery and cessation of NSAIDs remains the mainstay of management of this elusive condition.
